# Enzyme-Linked Phage Receptor Binding Protein Assays (ELPRA) Enable Identification of *Bacillus anthracis* Colonies

**DOI:** 10.3390/v13081462

**Published:** 2021-07-27

**Authors:** Peter Braun, Nadja Rupprich, Diana Neif, Gregor Grass

**Affiliations:** Department of Bacteriology and Toxinology, Bundeswehr Institute of Microbiology (IMB), 80937 Munich, Germany; peter3braun@bundeswehr.org (P.B.); nadjarupprich@googlemail.com (N.R.); diananeif@bundeswehr.org (D.N.)

**Keywords:** bacteriophage, receptor binding protein, reporter fusions, enzyme-linked phage protein assay, ELPRA, anthrax, *Bacillus anthracis*

## Abstract

Bacteriophage receptor binding proteins (RBPs) are employed by viruses to recognize specific surface structures on bacterial host cells. Recombinant RBPs have been utilized for detection of several pathogens, typically as fusions with reporter enzymes or fluorescent proteins. Identification of *Bacillus anthracis*, the etiological agent of anthrax, can be difficult because of the bacterium’s close relationship with other species of the *Bacillus cereus* *sensu lato* group. Here, we facilitated the identification of *B. anthracis* using two implementations of enzyme-linked phage receptor binding protein assays (ELPRA). We developed a single-tube centrifugation assay simplifying the rapid analysis of suspect colonies. A second assay enables identification of suspect colonies from mixed overgrown solid (agar) media derived from the complex matrix soil. Thus, these tests identified vegetative cells of *B. anthracis* with little processing time and may support or confirm pathogen detection by molecular methods such as polymerase chain reaction.

## 1. Introduction

Identification of *B. anthracis*, the etiological bacterial agent of anthrax disease of mammals, can be accomplished by bacteriophage (phage) sensitivity testing [1]. Phage-based specific detection of *B. anthracis* cells offers additional avenues for diagnostics of this notorious pathogen. Particularly, this complements nucleic acid-based detection techniques such as polymerase chain reaction (PCR), which is the current gold standard for *B. anthracis* identification [1]. Only a few phages have been found to be specific for *B. anthracis*. These mainly comprise phages Gamma [2,3], Wip1 [4], and AP50c [5]. Among these, phage Gamma is the one most widely used [2,3] and has a long history as a “diagnostic phage” [1,6]. A recent evaluation of its host specificity confirmed the Gamma phage’s suitability as its specificity reached 97% when tested against 700 aerobic, spore-forming bacteria, including other members of the closely related *Bacillus cereus sensu lato* group [7].

Host specificity of phages is typically determined by their receptor binding proteins (RBPs), which may be tail fibers or spike proteins. Typically, RBPs specifically recognize protein, teichoic acid, or polysaccharide entities on the host’s surface [8]. This interaction is the first step in the phage infection process. Recently, modified RBPs have opened new avenues for labeling, detecting, and capture of host bacterial cells. These phage RBP-based assays are already widely used as versatile tools for pathogen detection [9,10]. Target bacteria include biothreat agents that cause melioidosis (*Burkholderia pseudomallei*) [11], plague (*Yersinia pestis*) [12], or anthrax [13]. For *B. anthracis* phage Gamma, the GamR protein has been previously identified as the phage’s host cell receptor [14]. Recently, we have harnessed the Gamma phage RBP (Gp14) as a fluorescent reporter fusion for rapid microscopic detection of *B. anthracis* [13]. While functional, this reporter protein was difficult to heterologously produce in *Escherichia coli*. We thus resorted to a very similar protein, BA4079 [13], encoded by lambdoid prophage 03 located on the chromosome of *B. anthracis* [15]. BA4079, which acts as a specific *B. anthracis* RBP (named RBP_λ03_), and Gp14 (Gamma) share high amino acid sequence identities (83.0%; 89.0% similarity) with a continuous C-terminal region without gaps of 374 aa featuring 95.2% and 98.4% identity and similarity, respectively. An N-terminally truncated derivative of the BA4079 protein, termed RBP_λ03__Δ1-120_, was both highly soluble and bound specifically to *B. anthracis* cells over a broad range of growth phases [13]. As a fusion with mCherry, the fluorescent RBP reporter was used to identify *B. anthracis* cells via fluorescence microscopy. Specificity of RBP_λ03__Δ1-120_ toward *B. anthracis* was 95% (one false positive each among *B. cereus*, *B. weihenstephanensis*, and *B. paranthracis*) [13], thus, offering specificity quite similar to the 96–97% of phage Gamma [7].

In this study, we developed two enzyme-linked phage RBP assays (ELPRA) on the basis of the RBP_λ03__Δ1-120_ reporter as novel tools for identification of *B. anthracis.* This comprises a colony lift and blot ELPRA utilizing a luminogenic reporter fused to RBP_λ03__Δ1-120_, facilitating the detection of *B. anthracis* colonies after pre-enrichment from the complex matrix soil on solidified media. The alternative ELPRA implementation linking the RBP with a peroxidase function enabled rapid, colorimetric identification of live or inactivated colony material of *B. anthracis*.

## 2. Materials and Methods

### 2.1. Bacterial Culture, Soil Sample, B. anthracis Enrichment, and Cell Inactivation

Unless specified otherwise, *B. anthracis* Sterne [16] ATCC 4229 Pasteur and *B. cereus sensu lato* strains (Supplementary Materials Table S1) were grown on Columbia blood agar or *B. anthracis* agar [17]. A soil sample was taken from non-*B. anthracis*-contaminated park soil near the institute. *B. anthracis* Sterne was spiked in this soil as spores (generated according to [18], with modifications [13]) and enriched from this sample using a previously developed method [17]. Gamma phage sensitivity was tested by the melted overlay agar method [2]. Colonies of bacilli were chemically inactivated in aqueous peracetic acid solution (4% (*v*/*v*) Terralin PAA; Schülke & Mayr GmbH, Norderstedt, Germany), as described earlier [13].

### 2.2. DNA Isolation, Polymerase Chain Reaction, 16S rRNA Gene Sequencing, and Sequence Analysis

A bacterial colony grown on blood agar was chemically inactivated and DNA isolated using the MasterPure™ Gram Positive DNA Purification kit (Lucigen, Middleton, WI, USA) as described for Gram-positive bacteria, with minor modifications as described in [19]. DNA concentrations were quantified using the Qubit dsDNA HS Assay Kit (Thermo Fisher Scientific, Dreieich, Germany), according to the supplier’s protocol. DNA preparations were stored at −20 °C until further use.

For the identification of *B. anthracis* via PCR, the chromosomal marker *dhp61* was used as described previously [20]. The 16S rRNA gene region of new isolate *B. cereus s.l.* IMB-2021-1 was partially PCR-amplified using primer pairs 27r and 1492r [21] and subjected to DNA Sanger sequencing [21,22] (Eurofins Genomics Germany GmbH, Ebersberg, Germany).

### 2.3. Fluorescence Microscopy of Bacillus Cells Labeled with mCherry-RBP_λ03__Δ1-120_ Reporter

Chemically inactivated cells were labeled with mCherry-RBP_λ03__Δ1-120_ reporter protein, as described in [13]. In short, ca. 0.2 µg reporter protein was added to ca. 50 µL cells of an optical density at 600 nm (OD_600_) of 1 and 1 µL of the mixture was transferred into a well of a chamber slide with lid (µ-slide 8 Well, Ibidi GmbH, Martinsried, Germany). Cell suspensions were covered with a thin agarose pad and samples analyzed for mCherry signal (extinction: 587 nm, emission: 610 nm) using an Axio Observer Z1 700 Confocal Laser Scanning Microscope (Carl Zeiss, Oberkochen, Germany).

### 2.4. Cloning of a NanoLuc-RBP_λ03__Δ1-120_ Reporter Fusion Construct

For construction of an expression plasmid for heterologous production of TwinStrepTag (TST) tagged fusion protein NanoLuc-RBP_λ03__Δ1-120_, the previously generated plasmid pASG-IBA105*::tst::mCherry::RBP_λ03__Δ1-120_* was used as a basis [13]. Forward and reverse primers, used for amplification of the *NanoLuc* gene from template pNL1.1 (Promega, Walldorf, Germany), contained recognition sites for endonucleases BsrGI and XhoI, respectively (Supplementary Materials Table S2). These endonuclease recognition sites are also present up- and downstream of the *mCherry* gene in plasmid pASG-IBA105*::tst::mCherry::RBP_λ03__Δ1-120_* [13] and utilized to replace the *mCherry* gene with *NanoLuc*, resulting in plasmid pASG-IBA105::*tst*::*NanoLuc*::*RBP_λ03__Δ1-120_*.

### 2.5. Expression, Purification, and Western Blot Analysis of Strep-Tagged NanoLuc/mCherry-RBP_λ03__Δ1-120_ Reporter Fusions

The pASG-IBA105::*tst*::*NanoLuc*::*RBP_λ03__Δ1-120_* and the pASG-IBA105::*tst*::*mCherry*::*RBP_λ03__Δ1-120_* plasmids were transformed into *E. coli* ArcticExpress cells (Agilent Technologies Inc., Waldbronn, Germany). A single colony was used for protein production, as described in [13]. In short, for protein production, an exponentially growing culture at an optical density of 0.6–0.8 (OD_600_) was cooled down to 12 °C, induced with anhydrotetracycline, and incubation continued for 24 h at 12 °C. Cells were harvested, lysed, and filtered. The filtered lysate was subjected to affinity chromatography (Äkta pure system; GE Healthcare Life Science, Munich, Germany) using a 1 mL Strep-Tactin^®^ XT column (IBA GmbH, Göttingen, Germany). The eluted protein was dialyzed against HEPES buffer (50 mM HEPES, 50 mM NaCl, 5 mM EDTA, pH 7.5), protein concentration measured (Pierce BCA Protein Assay Kit; ThermoFisher Scientific, Darmstadt, Germany), and adjusted to a concentrations of 1 mg protein/mL. Protein aliquots were either kept at −80 °C for long-term storage use or mixed with 50% (*v*/*v*) glycerol (final concentration) as a cryo-protectant and stored at −20 °C for testing in RBP-fusion reporter assays.

SDS-PAGE and Western blot analysis was performed as described in [13]. The polyacrylamide gel (Novex NuPAGE 4–12% Bis-Tris protein-gel; ThermoFisher Scientific, Darmstadt, Germany) was transferred onto a 0.45 µm pore size nitrocellulose membrane (ThermoFisher Scientific, Darmstadt, Germany) and subjected to semi-dry blotting at 30 V for 75 min (Novex Semi-Dry Blotter, ThermoFisher Scientific, Darmstadt, Germany). TST-tagged proteins were detected using Strep-MAB-Classic (HRP antibody conjugate, IBA GmbH, Göttingen, Germany) via chemiluminescence detection (Clarity Western ECL substrate; Bio-Rad Laboratories, Munich, Germany), according to the manufacturers’ protocols.

### 2.6. Horseradish Peroxidase Labeling of mCherry-RBP_λ03__Δ1-120_ Fusion Protein

The mCherry-RBP_λ03__Δ1-120_ fusion protein was labeled with horseradish peroxidase (HRP) using the EZ-Link™ Plus Activated Peroxidase Kit (ThermoFisher Scientific, Darmstadt, Germany) in carbonate–bicarbonate buffer (pH 9.4), according to the manufacturer’s protocol.

### 2.7. Colony Lift and Blot ELPRA for NanoLuc -RBP_λ03__Δ1-120_ Reporter-Mediated Detection and Identification of B. anthracis

Agar plates from enrichment grown overnight were blotted onto hydrophobic nitrocellulose membranes (Carl Roth, Karlsruhe, Germany). For this, membranes were cut into circles fitting into 81 mm diameter plastic petri dishes using a home-made cardboard template. The membrane was labeled with a permanent pen as was the corresponding rim of the petri dish to ensure reconstruction of the relative orientation of plate and membrane. The colony lift method was loosely adapted from [23], the colony blot assay modified from manual protocols “Strep-tag^®^ detection in Western blots” (chapter 2; IBA GmbH, Göttingen, Germany) and “Nano-Glo^®^ HiBiT Blotting System” (chapter 3; Promega, Walldorf, Germany). The colony lift and blot comprised the following steps: a membrane was carefully lowered onto the agar surface and softly pressed so complete contact between membrane and agar (colonies) was achieved (air bubbles can escape). The membrane was immediately (<10 s) removed using forceps (“lift”) and carefully pressed onto a pre-wetted (with blocking buffer, i.e., 3% (*w*/*v*) bovine serum albumin (BSA) in Tris-buffered saline (TBS, pH 7.4) thick Whatman filter paper (Life Technologies, Darmstadt, Germany) with the colony-bearing side down to remove superfluous colony material not yet attached to the membrane. The membrane was carefully lifted and immediately (without drying) submerged into 15 mL blocking buffer (in an unused petri dish) and rocked gently for 30 min in order to block unspecific binding sites on the membrane. The blocking buffer was replaced with 20 mL TBS wash buffer. After about one minute with light agitation, the wash buffer was replaced with 5 mL TBST (TBS with 0.05% (*v*/*v*) Tween 20) containing 0.2 µg NanoLuc-RBP_λ03__Δ1-120_ and the petri dish was gently rocked for 10 min to facilitate RBP binding to membrane-attached cells. The membrane (in petri dish) was then washed four times with 15 mL fresh TBST with gentle agitation for about 1 min. In the meantime, 7.5 mL Nano-Glo^®^-Blotting-Buffer (from Nano-Glo^®^ HiBiT Blotting System, Promega, Walldorf, Germany) was diluted to 1x (from 10x stock) with sterile aqua_dest_. To yield NanoLuc substrate buffer, the 1x blotting buffer was mixed with 15 μL Nano-Glo^®^ Luciferase Assay Substrate (from Nano-Glo^®^ HiBiT Blotting System, Promega, Walldorf, Germany) and poured into a fresh petri dish. The membrane was dipped and completely submerged into NanoLuc substrate buffer from both sides (“blot”) and transferred immediately (without drying) onto a transparent plastic foil with the colony-bearing side up. The membrane was covered with a second foil and transferred into a suitable transparent transport container. Luminescence was recorded on a ChemiDoc MP imaging system (Bio-Rad Laboratories, Munich, Germany) with Image Lab 5.2 software (Bio-Rad Laboratories, Munich, Germany) for documentation.

### 2.8. Rapid Dichotomous Colorimetric ELPRA for Identification of Suspect B. anthracis Colonies

To identify a suspect *B. anthracis* colony, it was lifted with a loop and resuspended into a 1.5 mL reaction tube containing 100 µL PBS. From this, up to 50 µL was transferred to a new tube and 50 µL blocking buffer (3% (*w*/*v*) BSA in phosphate-buffered saline) was added. For two-step, indirect ELPRA, 0.2 µg of mCherry-RBP_λ03__Δ1-120_ reporter (fluorescence of mCherry is irrelevant here, any protein featuring a TST can be used) was added and the reaction was either flicked by hand a couple of times or shaken at 600 rpm for 1 min. Next, 1 mL PBST (PBS with 0.05% Tween 20) was added, mixed and centrifuged for 1 min at 10,000× *g*. The pellet was resuspended in 1.5 mL PBST and pelleted by centrifugation at 10,000× *g*. Strep-Tactin^®^ horse radish peroxidase conjugate (IBA GmbH, Göttingen, Germany) was diluted 1:4000 into PBST and 100 µL used to resuspend the cell pellet. The sample was either flicked by hand a couple of times or shaken at 600 rpm for 1 min. One mL PBST was added, mixed and centrifuged for 1 min at 10,000× *g*. The pellet was washed once with 1.5 mL PBST and once with 1.5 mL PBS. Finally, the pellet was resuspended in 50 µL SeramunBlau^®^ slow (containing 3,3′,5,5′-tetramethylbenzidin) peroxidase substrate (Seramun Diagnostica GmbH, Heidesee, Germany). Blue color development was monitored for several minutes and photo-documented (photos were adjusted for contrast and brightness). If necessary, the color reaction was stopped by centrifugation and removal of the cell pellet.

Alternatively, 0.1 µg HRP-conjugated mCherry-RBP_λ03__Δ1-120_ reporter was used for one-step ELPRA, replacing separate steps of RBP and HRP addition to colony material. All other incubation and wash steps were the same as described for the two-step ELPRA. All steps were conducted at room temperature. As process controls served *B. anthracis* colony material treated as above but (i) TST-tagged protein, (ii) peroxidase conjugate, or (iii) both were replaced with PBS. Colony material of *B. cereus* served as negative control.

Colony material of any *B. anthracis* strain may be used as positive control when assaying suspect colonies. This assay may be conducted using live or inactivated cells of *B. anthracis* or *B. cereus s.l.* (controls). Complete inactivation of *B. anthracis* cells and spores was achieved using 4% (*v*/*v*) Terralin PAA [13]. Other means of inactivation may also work for this assay to varying degrees [13].

## 3. Results

### 3.1. Production of the Recombinant Luminescence-Reporter NanoLuc-RBP_λ03__Δ1-120_

We adapted the colony lift and colony blot techniques for the detection and identification of *B. anthracis* on solidified (agar) media. Initial experiments using our established mCherry-RBP_λ03__Δ1-120_ reporter (pASG-IBA105::*tst*::*mCherry*::*RBP_λ03__Δ1-120_*) featuring a TST epitope [13] for detection of *B. anthracis* colonies by colony lift and blot assay yielded unsatisfactory results. Discrimination between signal (*B. anthracis* colonies) and background (bacteria-loaded membrane) was poor when using horseradish peroxidase as a reporter. We thus resorted to using the visible light generating reporter protein NanoLuc, a truncated derivative of deep-sea shrimp (*Oplophorus gracilirostris*) luciferase. The heterologously produced NanoLuc-RBP_λ03__Δ1-120_ reporter protein was soluble but appeared slightly smaller on SDS-PAGE than the expected molecular weight of 66 KDa, as shown in Supplementary Materials Figure S1. The yield was about 5 mg protein/L culture.

### 3.2. A Colony Lift and Luminescent Blot-Based ELPRA Using NanoLuc-RBP_λ03__Δ1-120_ as Reporter Probe Facilitates Identification of B. anthracis

**Figure 1:**
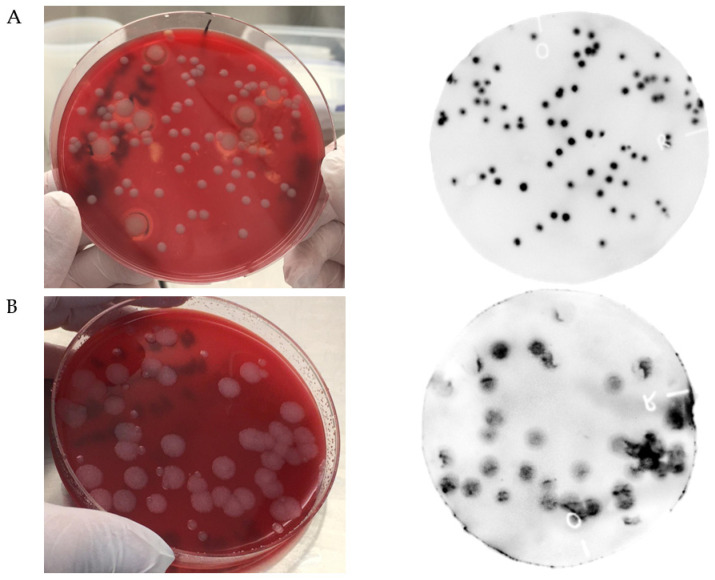
Luminogenic reporter probe NanoLuc-RBP_λ03__Δ1-120_ for differentiation of *B. anthracis* from *B. cereus* in a colony lift and blot assay. Cells of *B. anthracis* and *B. cereus* were mixed and plated on solid anthrax blood agar media. (**A**) *B. anthracis* Sterne and *B. cereus* ATCC 10,987 grown overnight at 28 °C; (**B**) *B. anthracis* Sterne and *B. cereus* environmental isolate IMB-4-0-Rott grown for 24 h at 37 °C. Left panels: photos of incubated agar plates; right panels: luminescence signals on nitrocellulose membranes after lift and blot assay from respective agar plate. Markings (“O” and “R) are just for orientation and alignment.

As a proof of principle for detection of *B. anthracis* colonies, the NanoLuc-RBP_λ03__Δ1-120_ reporter served as a luminescence-generating probe. Figure 1A shows the result of detecting colonies of *B. anthracis* in a mixed culture plate with *B. cereus* ATCC10987. All membrane-transferred *B. anthracis* colonies but none of the *B. cereus* colonies showed significant luminescence resulting from the specific binding of NanoLuc-RBP_λ03__Δ1-120_ to the *B. anthracis* cells. Starting from the colony lift step, the assay takes about 1.5–2 h until completion.

Since differentiation between typical *B. anthracis-* and *B. cereus*-colonies on an erythrocyte-containing agar plate is obvious due to the hemolysis exerted by the bigger *B. cereus* colonies, we next tested the assay against an environmental, non-hemolytic *B. cereus* isolate (strain IMB-4-0-Rott) that forms colonies on anthrax blood agar that look suspiciously similar to *B. anthracis*. To increase the visual confusion, we prolonged the incubation time to 24 h at 37 °C, after which *B. anthracis* formed large colonies resembling that of typical strains of non-hemolytic *B. cereus,* whereas the non-hemolytic *B. cereus* strain formed small colonies (Figure 1B, left panel). Indeed, luminescence probe-based detection identified the correct, i.e., large-sized colonies (Figure 1B, right panel).

### 3.3. B. anthracis Can Be Detected and Identified from Spiked Soil Sample Preparations Using Colony Lift and Blot Based ELPRA with NanoLuc-RBP_λ03__Δ1-120_ as Reporter Probe

**Figure 2:**
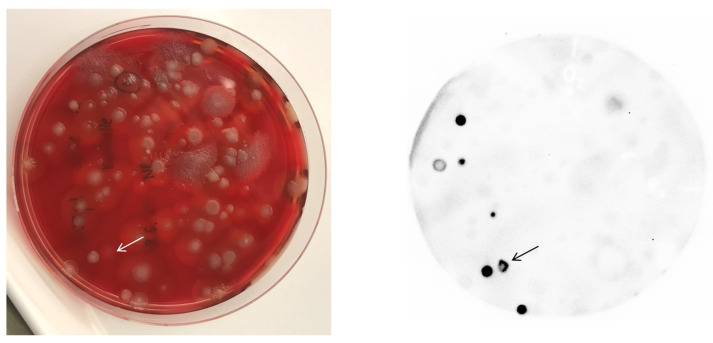
Colony lift and blot assay with luminogenic reporter probe NanoLuc-RBP_λ03__Δ1-120_ for identification of *B. anthracis* in a heterogeneous environmental plate culture. A soil sample was spiked with *B. anthracis* spores, subjected to enrichment, plated on *B. anthracis*-agar [17], and assayed by lift and blot ELPRA for *B. anthracis*. Left panel: photo of incubated agar plate; right panel: luminescence signals on nitrocellulose membrane after lift and blot ELPRA from respective agar plate. The colony labeled with an arrow showing untypical colony morphology for *B. anthracis* was selected for further analysis.

Enriching and isolating *B. anthracis* from complex environmental matrices can be challenging. This becomes a nuisance with low *B. anthracis* spore concentrations in soil samples in the presence of a relative high abundance of related bacilli and other spore formers. Therefore, we combined semi-selective enrichment of *B. anthracis* from soil on solid agar medium [17] with the new colony lift and blot assay. For lack of authentic soil-samples contaminated with *B. anthracis*, we spiked *B. anthracis*-free soil samples with spores of *B. anthracis* prior to enrichment. Colonies from overgrown plates were then lifted and blotted. As shown in Figure 2, individual *B. anthracis* colonies can easily be identified on the membrane. The corresponding location on the agar plate can be deducted by comparing the photo of the overgrowing plate (Figure 2, left panel) with the photo of the developed colony bearing membrane (Figure 2, right panel). From there, it should be straight forward to re-streak colony material from this plate area to a fresh plate and to further test arising suspect individual colonies.

We did so for one colony giving rise to signals in Figure 2, (arrow). Its colony morphology resembled *Bacillus mycoides* rather than *B. anthracis*. After subculture on a fresh agar plate, this fuzzy phenotype remained. The isolate was sensitive to Gamma as the phage produced plaques on pour plates [2], but negative for the *B. anthracis* PCR marker *dhp61* [20]. Sequencing of the isolate’s partial 16S rRNA gene revealed that this bacterium, which was named IMB-2021-1, had as closest characterized relatives (with identical DNA sequences over 1462 bp in the 16S rRNA gene): *Bacillus toyonensis* strain MCCC 1A00418 (GenBank: KJ812421), *Bacillus toyonensis* strain MCCC 1A01056 (GenBank: KJ812432), and *Bacillus wiedmannii* strain SX13.1LB (GenBank: MT052668). Thus, strain IMB-2021-1 very likely represented a new *B. toyonensis* or *B. wiedmannii* strain.

### 3.4. Suspect B. anthracis Colonies Can Be Identified by ELPRA Using Strep-Tagged-RBP_λ03__Δ1-120_ Derivatives as a Dichotomous Colorimetric Reporter

**Figure 3:**
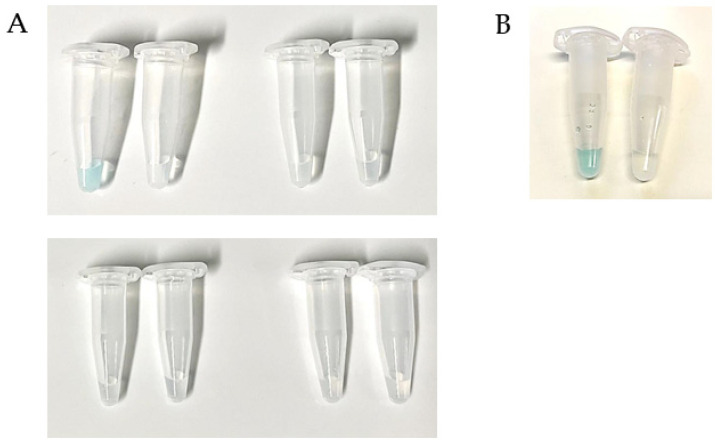
Two-step dichotomous colorimetric centrifugation ELPRA for RBP-dependent identification of *B. anthracis* cells. (**A**) Live colony material (50 µL of ca. 0.5 OD_600_) of *B. anthracis* Sterne (left sample in each pair) or *B. cereus* ATCC10987 (right sample in each pair) were first labeled with RBP_λ03__Δ1-120_ reporter probe (step 1). After several buffer washes, Strep-Tactin^®^-HRP conjugate was added to cell solutions (step 2). Cells were washed again with buffer and chromogenic substrate was added, which is converted by HRP into a blue dye. From left to right, first row: Complete assay (steps 1 + 2); process control 1 (step 2 only, i.e., no RBP probe); second row: process control 2 (step 1 only, i.e., no Strep-Tactin^®^-HRP-conjugate); process control 3 (neither step 1 nor step 2). (**B**) Same as (**A**, first pair) but inactivated cell material was used.

The TST-labeled mCherry-RBP_λ03__Δ1-120_ reporter has previously facilitated rapid identification via fluorescent microscopy within a few minutes [13]. In an effort to make this assay more accessible to laboratories lacking sophisticated equipment, we designed a rapid dichotomous (“yes/no”) colorimetric test. The mCherry component of the reporter construct does not participate in signal generation but instead serves to enhance solubility of the heterologous protein and facilitates monitoring of protein production and purification. In a test tube, material from a single suspect colony is successively mixed with the reporter RBP harboring a TST epitope, a Strep-Tactin^®^–horseradish–peroxidase conjugate (Strep-Tactin^®^-HRP) and chromogenic HRP substrate. Samples containing *B. anthracis* colony material turn blue because the RBP reporter binds to the cell surfaces, the attached TST is recognized and binds to Strep-Tactin^®^-HRP, which in turn oxidizes the chromogenic substrate. This assay, including wash steps, was optimized for speed and can be completed within <30 min. A representative test is shown in Figure 3A. Clearly, the sample containing *B. anthracis* cell material turned blue, whereas the sample with *B. cereus* remained colorless (as did several process controls). This assay works with both live and peracetic acid inactivated cells (Figure 3B), giving flexibility to perform the assay within or outside BSL-3 containment.

### 3.5. The Dichotomous Colorimetric ELPRA for Identification of B. anthracis Can Be Simplified to a One-Step Assay

**Figure 4:**
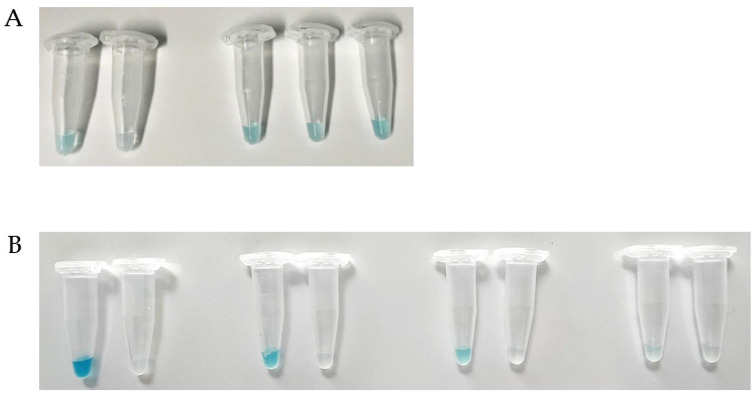
Identification of *B. anthracis* cells by rapid one-step dichotomous colorimetric ELPRA. (**A**) Inactivated colony material (50 µL of ca. 0.5 OD_600_) of *B. anthracis* Sterne, *B. cereus* ATCC10987, *B. cereus s.l.* IMB-2021-1, *B. cereus* CDC2000032805, or *B. cereus* ATCC4342 (from left to right) were labeled in a one-step reaction with Strep-Tactin^®^XT-HRP-conjugated RBP_λ03__Δ1-120_ reporter probe, washed several times with buffer, and chromogenic substrate was added, which was converted by HRP to a blue dye. (**B**) Same as (**A**) but a 1:5 dilution series is shown for *B. anthracis* Sterne and *B. cereus* ATCC10987 using 50 µL of colony material. The second pair of tubes from the left was taken from cell material of an optical density of 1 (OD_600_).

Alternative to the two-step ELPRA approach (RBP binding followed by Strep-Tactin^®^-HRP binding to cells) described above, we also developed a one-step test system. For this, the HRP moiety was directly conjugated to the RBP_λ03__Δ1-120_ reporter protein. When HRP-RBP_λ03__Δ1-120_ was tested on inactivated *B. anthracis* Sterne and *B. cereus* ATCC10987 colony material, only *B. anthracis* yielded blue signals (Figure 4A).

While the one-step ELPRA assay required additional work beforehand and financial investment imposed by the RBP-Strep-Tactin^®^-HRP conjugation procedure, it further sped up the entire assay process and diminished pipetting steps. As a consequence, three parallel samples (an unknown sample and positive and negative control colony material) can be processed in as little as 20 min until scoring results (blue vs. no color).

Since there are a few *B. cereus s.l.* isolates known to yield false-positive results for binding of RBP_λ03__Δ1-120_ [13] and to serve as host for Gamma phage as well [7], we included two of such strains in our testing. Additionally, we included the new isolate *B. cereus s.l.* IMB-2021-1 recovered by the colony lift and blot assay from Figure 2. Inactivated colony material of both *B. cereus* 4342 and CDC2000032805 was recognized by the HRP-RBP_λ03__Δ1-120_ reporter, as indicated by blue color development (Figure 4A). Similarly, the new isolate *B. cereus s.l.* IMB-2021-1 was also receptive for the RBP reporter. This finding was corroborated by the Gamma phage assay [2] and fluorescence microscopy using the mCherry-RBP_λ03__Δ1-120_ reporter (lacking conjugated HRP; Supplementary Materials Figure S2) vis-à-vis *B. anthracis* in which *B. cereus* 4342 and CDC2000032805 showed at least partially labeled cells. The partial labeling may also explain the lighter blue color of samples containing these isolates compared to cells of *B. anthracis*. Notably, isolate *B. cereus s.l.* IMB-2021-1 cells became fluorescent to the same extent as *B. anthracis* and the labeling reaction in Figure 4A commenced with a similar velocity.

In order to determine cell concentration ranges suitable for one-step dichotomous colorimetric ELPRA, we assayed a 1:5 dilution series of inactivated *B. anthracis* or *B. cereus* cell material. Dilutions were adjusted, so the first 1:5 dilution step samples had optical densities of 1 (OD_600_) (Figure 4B; second pair of tubes). While the 1:125 dilution contained too little cell material to elicit any signal from *B. anthracis* cells, the 1:25 dilution was sufficient to differentiate the *B. anthracis* signal from that of *B. cereus* (no signal). A strong signal developed from the 1:5 (OD 1) sample and the undiluted sample produced a very strong signal. At all these cell densities, *B. cereus* still did not yield any visible signal (Figure 4B). Notably, the signal from undiluted *B. anthracis* sample arose very rapidly, within a few seconds after addition of chromogenic substrate to the sample. Other signals took a couple minutes to develop. These results indicate that the one-step dichotomous colorimetric ELPRA for identification of *B. anthracis* is both as specific as the Gamma phage assay, largely flexible to the amount of cell material used, and quick to perform.

## 4. Discussion

Detection and identification of *B. anthracis* faces a variety of challenges. First, vegetative cells and spores may not be readily susceptible to identical analytical techniques. Second, *B. anthracis* is notoriously difficult to differentiate from its closest neighbors of the *B. cereus s.l.* group. The Gold Standard for *B. anthracis* identification still remains PCR targeting species-specific genetic markers such as *dhp61* [20], *PL3* [24], or others, including single nucleotide variations in genes like *plcR* [25]. Less reliable methods than PCR for *B. anthracis* identification are also available that may confirm PCR results or serve as rapid preliminary screening tools for pathogen detection. A common approach, especially in the field or in mobile laboratory settings, is the use of lateral flow assays (LFAs) for their quick and easy application. Unfortunately, LFAs are repeatedly neither very sensitive (in terms of limit of detection) nor highly specific [26,27]. For instance, a well-documented validation of the Tetracore RedLine Alert LFA yielded a sensitivity of >97% for *B. anthracis* with only a single *B. cereus* giving a false-positive result. However, upon closer examination, the panel of organisms tested included only seven *B. cereus* isolates [28]. In contrast, application of RBP_λ03__Δ1-120_ for microscopy-based identification of *B. anthracis* has previously shown 95% specificity with only three false-positive *B. cereus s.l.* out of 56 non-*B. anthracis* bacilli. Similarly, another commercial LFA (InBios Active Anthrax Detect Rapid Test) reached 82% specificity [3], however, samples used in that study also included more difficult to test contaminated animal tissues. In contrast to many RBP-based assays [12,13], however, most LFAs are not depending on samples derived from actively growing *B. anthracis* cells. Among the RBPs previously tested for *B. anthracis*, we selected RBP_λ03__Δ1-120_ because it is both host-specific and the least affected by the growth phases of its host cells [13]. Notably, for the intended use of screening of fresh growth on agar (colony lift and blot ELPRA) or of colony material (colorimetric ELPRA), growth-phase dependency of the RBP may not need to be a limiting factor. Previously, we have shown that RBP_λ03__Δ1-120_ is able to label encapsulated cells of *B. anthracis* [13]. Though we did not explicitly retest this again, we expect the rapid assay introduced here works on encapsulated cells as well. Conversely, some LFAs have limitations, e.g., LFAs for *B. anthracis* detection requiring spores rather than cells as targets may fail to detect vegetative cells [27]. Conversely, ELPRA using RBP_λ03__Δ1-120_ does not recognize carefully purified spores (i.e., preparations devoid of dead cells, ghosts, and cell debris) at all [13].

Spectrometric methods require specialized instrumentation and typically highly trained personnel [29,30]. In contrast, the long-established Gamma phage assay has proven to be a useful tool to complement PCR-based *B. anthracis* identification as it is both cheap and does not require specialized equipment or training [1]. With a high specificity of 96–97% toward *B. anthracis* [7], its usefulness is only limited by the duration of the identification procedure, which requires actively growing cells of *B. anthracis* [6]. In this the Gamma phage assay is similar to the ELPRA, but RBP_λ03__Δ1-120_ is less restricted to the growth phase [13].

The *B. anthracis*-specific RBPs reported in this and previous work [13] bridge host-specific phage-based identification and ease of application with the speed of the detection assay. Starting from colony material, results can be obtained in just a few minutes using the one-step dichotomous colorimetric assay introduced here. Of course, another limit inherent in the Gamma phage assay carries over to the advanced RBP assay. Specificity remains high (>95%) but does not reach the near 100% certainty of PCR tests [20,24,25].

Colony blot assays for detecting microorganisms have a long history of application. They can help detect colonies of rare target organisms among those of other species isolated from complex sample matrices, especially when there are no suitable means of prior enrichment. While antibodies or sera are typically recruited for primary detection of the target organism, e.g., [23,31], subsequent enrichment by cultivating colony-bearing membranes can also be used [32]. Alternatively, DNA probes may be employed on lysed target cells if genetic material is assayed, e.g., [33]. A phage-derived RBP, as in the study at hand, has, to the best of our knowledge, not yet been used for the purpose of target bacteria detection by colony blot before.

The newly introduced colony lift and blot ELPRA for *B. anthracis* may not only be useful for the analysis of complex environmental matrices with only minute numbers of contaminating *B. anthracis*. If extant semi-selective growth media overwhelmed by overgrowing bacterial flora are used, positions of positive signals on such agar plates can still be located and subjected to further analysis. Possibly, future progress in further developing *B. anthracis*-specific agar media may ameliorate this issue. Recently, for instance, a new selective agar medium for *B. anthracis* has been introduced [34]. The colony lift and blot ELPRA may be applied in conjunction with this improved agar medium for complex environmental samples bearing only low contamination of *B. anthracis* spores. Alternatively or in combination, the ELPRA could be included as a final step of analysis of more complex spore-enrichment procedures such as the one described in [35]. Herein, very low spore contaminations in soil of only 14 *B. anthracis* spores per g soil could be detected; however, the enrichment procedure took about 3.5 h (excluding cultivation). For the lack of authentic soil samples contaminated with *B. anthracis*, we were not able to compare our colony lift and blot ELPRA against this earlier protocol. Possibly, the colony lift and blot ELPRA may be combined with immunomagnetic enrichment for capturing *B. anthracis* spores [36,37]. Additionally, the colony lift and blot ELPRA for *B. anthracis* may also become a tool for the detection and subsequent isolation of bacteria outside *B. anthracis* that have properties that enabled these host cells to sequester RBPs or even complete phages.

## 5. Conclusions

This study introduced two new RBP-based identification assays for *B. anthracis*. The colony lift and blot ELPRA can be expected to facilitate *B. anthracis* identification from complex environmental matrices. The rapid colorimetric ELPRA may support PCR-based testing, for example, by pre-screening of suspect colonies for *B. anthracis*. More generally, RBP derivatives provide a valuable extension to the toolbox for pathogen detection and are both relatively easy to produce and to adapt to newly arising diagnostic needs.

## Supplementary Materials

The following are available online at https://www.mdpi.com/article/10.3390/v13081462/s1, Figure S1: Western blot of heterologously produced NanoLuc-RBP_λ03Δ1-120_ reporter protein, Figure S2: Binding of mCherry-RBP_λ03Δ1-120_ reporter to cells of cultures of B. anthracis and cross-reacting B. cereus cells, Table S1: Strains used in this work, Table S2: Oligonucleotide primers used for DNA sequencing and cloning in this work.

## References

[1] Turnbull P., World Health Organization (2008). Anthrax in Humans and Animals Fourth Edition.

[2] Abshire T.G., Brown J.E., Ezzell J.W. (2005). Production and Validation of the Use of Gamma Phage for Identification of Bacillus Anthracis. J. Clin. Microbiol..

[3] Kolton C.B., Marston C.K., Stoddard R.A., Cossaboom C., Salzer J.S., Kozel T.R., Gates-Hollingsworth M.A., Cleveland C.A., Thompson A.T., Dalton M.F. (2019). Detection of *Bacillus anthracis* in animal tissues using InBios active anthrax detect rapid test lateral flow immunoassay. Lett. Appl. Microbiol..

[4] Kan S., Fornelos N., Schuch R., Fischetti V.A. (2013). Identification of a ligand on the Wip1 bacteriophage highly specific for a receptor on *Bacillus anthracis*. J. Bacteriol..

[5] Sozhamannan S., McKinstry M., Lentz S.M., Jalasvuori M., McAfee F., Smith A., Dabbs J., Ackermann H.W., Bamford J.K., Mateczun A. (2008). Molecular characterization of a variant of *Bacillus anthracis*-specific phage AP50 with improved bacteriolytic activity. Appl. Environ. Microbiol..

[6] Brown E.R., Cherry W.B. (1955). Specific identification of *Bacillus anthracis* by means of a variant bacteriophage. J. Infect. Dis..

[7] Kolton C.B., Podnecky N.L., Shadomy S.V., Gee J.E., Hoffmaster A.R. (2017). *Bacillus anthracis* gamma phage lysis among soil bacteria: An update on test specificity. BMC Res. Notes.

[8] Dowah A.S.A., Clokie M.R.J. (2018). Review of the nature, diversity and structure of bacteriophage receptor binding proteins that target Gram-positive bacteria. Biophys. Rev..

[9] Dunne M., Loessner M.J. (2019). Modified bacteriophage tail fiber proteins for labeling, immobilization, capture, and detection of bacteria. Methods Mol. Biol..

[10] Denyes J.M., Dunne M., Steiner S., Mittelviefhaus M., Weiss A., Schmidt H., Klumpp J., Loessner M.J. (2017). Modified bacteriophage S16 long tail fiber proteins for rapid and specific immobilization and detection of *Salmonella* cells. Appl. Environ. Microbiol..

[11] Muangsombut V., Withatanung P., Chantratita N., Chareonsudjai S., Lim J., Galyov E.E., Ottiwet O., Sengyee S., Janesomboon S., Loessner M.J. (2021). Development of a bacteriophage tail fiber-based latex agglutination assay for rapid clinical screening of *Burkholderia pseudomallei*. Appl. Environ. Microbiol..

[12] Born F., Braun P., Scholz H.C., Grass G. (2020). Specific detection of *Yersinia pestis* based on receptor binding proteins of phages. Pathogens.

[13] Braun P., Wolfschläger I., Reetz L., Bachstein L., Jacinto A.C., Tocantins C., Poppe J., Grass G. (2020). Rapid microscopic detection of *Bacillus anthracis* by fluorescent receptor binding proteins of bacteriophages. Microorganisms.

[14] Davison S., Couture-Tosi E., Candela T., Mock M., Fouet A. (2005). Identification of the *Bacillus anthracis* (gamma) phage receptor. J. Bacteriol..

[15] Sozhamannan S., Chute M.D., McAfee F.D., Fouts D.E., Akmal A., Galloway D.R., Mateczun A., Baillie L.W., Read T.D. (2006). The *Bacillus anthracis* chromosome contains four conserved, excision-proficient, putative prophages. BMC Microbiol..

[16] Sterne M., Proom H. (1957). Induction of motility and capsulation in *Bacillus anthracis*. J. Bacteriol..

[17] Fasanella A., Di Taranto P., Garofolo G., Colao V., Marino L., Buonavoglia D., Pedarra C., Adone R., Hugh-Jones M. (2013). Ground Anthrax *Bacillus* Refined Isolation (GABRI) method for analyzing environmental samples with low levels of *Bacillus anthracis* contamination. BMC Microbiol..

[18] Cote C.K., Buhr T., Bernhards C.B., Bohmke M.D., Calm A.M., Esteban-Trexler J.S., Hunter M., Katoski S.E., Kennihan N., Klimko C.P. (2018). A standard method to inactivate *Bacillus anthracis* spores to sterility via gamma irradiation. Appl. Environ. Microbiol..

[19] Knüpfer M., Braun P., Baumann K., Rehn A., Antwerpen M., Grass G., Wölfel A.R. (2020). Evaluation of a highly efficient DNA extraction method for *Bacillus anthracis* endospores. Microorganisms.

[20] Antwerpen M.H., Zimmermann P., Bewley K., Frangoulidis D., Meyer H. (2008). Real-time PCR system targeting a chromosomal marker specific for *Bacillus anthracis*. Mol. Cell. Probes.

[21] Lane D.J., Pace B., Olsen G.J., Stahl D.A., Sogin M.L., Pace N.R. (1985). Rapid determination of 16S ribosomal RNA sequences for phylogenetic analyses. Proc. Natl. Acad. Sci. USA.

[22] Watanabe K., Kodama Y., Harayama S. (2001). Design and evaluation of PCR primers to amplify bacterial 16S ribosomal DNA fragments used for community fingerprinting. J. Microbiol. Methods.

[23] Bogaert D., Veenhoven R.H., Sluijter M., Sanders E.A., de Groot R., Hermans P.W. (2004). Colony blot assay: A useful method to detect multiple pneumococcal serotypes within clinical specimens. FEMS Immunol. Med. Microbiol..

[24] Wielinga P.R., Hamidjaja R.A., Agren J., Knutsson R., Segerman B., Fricker M., Ehling-Schulz M., de Groot A., Burton J., Brooks T. (2011). A multiplex real-time PCR for identifying and differentiating *B. anthracis* virulent types. Int. J. Food. Microbiol..

[25] Derzelle S., Mendy C., Laroche S., Madani N. (2011). Use of high-resolution melting and melting temperature-shift assays for specific detection and identification of *Bacillus anthracis* based on single nucleotide discrimination. J. Microbiol. Methods.

[26] Zasada A.A., Forminska K., Zacharczuk K., Jacob D., Grunow R. (2015). Comparison of eleven commercially available rapid tests for detection of *Bacillus anthracis*, *Francisella tularensis* and *Yersinia pestis*. Lett. Appl. Microbiol..

[27] Ziegler I., Vollmar P., Knüpfer M., Braun P., Stoecker K. (2021). Reevaluating limits of detection of 12 lateral flow immunoassays for the detection of *Yersinia pestis*, *Francisella tularensis*, and *Bacillus anthracis* spores using viable risk group-3 strains. J. Appl. Microbiol..

[28] Pillai S.P., Prentice K.W., Ramage J.G., DePalma L., Sarwar J., Parameswaran N., Bell M., Plummer A., Santos A., Singh A. (2019). Rapid presumptive identification of *Bacillus anthracis* isolates using the Tetracore RedLine Alert Test. Health. Secur..

[29] Dybwad M., van der Laaken A.L., Blatny J.M., Paauw A. (2013). Rapid identification of *Bacillus anthracis* spores in suspicious powder samples by using matrix-assisted laser desorption ionization-time of flight mass spectrometry (MALDI-TOF MS). Appl. Environ. Microbiol..

[30] Pauker V.I., Thoma B.R., Grass G., Bleichert P., Hanczaruk M., Zoller L., Zange S. (2018). Improved discrimination of *Bacillus anthracis* from closely related species in the *Bacillus cereus sensu lato* group based on Matrix-Assisted Laser Desorption Ionization-Time of Flight mass spectrometry. J. Clin. Microbiol..

[31] Roop R.M., Preston-Moore D., Bagchi T., Schurig G.G. (1987). Rapid identification of smooth *Brucella* species with a monoclonal antibody. J. Clin. Microbiol..

[32] Hoszowski A., Fraser A.D., Brooks B.W., Riche E.M. (1996). Rapid detection and enumeration of *Salmonella* in chicken carcass rinses using filtration, enrichment and colony blot immunoassay. Int. J. Food. Microbiol..

[33] Ramotar K., Henderson E., Szumski R., Louie T.J. (1995). Impact of free verotoxin testing on epidemiology of diarrhea caused by verotoxin-producing *Escherichia coli*. J. Clin. Microbiol..

[34] Rohde A., Papp S., Feige P., Grunow R., Kaspari O. (2020). Development of a novel selective agar for the isolation and detection of *Bacillus anthracis*. J. Appl. Microbiol..

[35] Silvestri E.E., Feldhake D., Griffin D., Lisle J., Nichols T.L., Shah S.R., Pemberton A., Schaefer F.W. (2016). Optimization of sample processing protocol for recovery of *Bacillus anthracis* spores from soil. J. Microbiol. Methods.

[36] Shields M.J., Hahn K.R., Janzen T.W., Goji N., Thomas M.C., Kingombe C.B., Paquet C., Kell A.J., Amoako K.K. (2012). Immunomagnetic capture of *Bacillus anthracis* spores from food. J. Food. Prot..

[37] Fisher M., Atiya-Nasagi Y., Simon I., Gordin M., Mechaly A., Yitzhaki S. (2009). A combined immunomagnetic separation and lateral flow method for a sensitive on-site detection of *Bacillus anthracis* spores—Assessment in water and dairy products. Lett. Appl. Microbiol..

