# Supplementary material for: Enzyme-Linked Phage Receptor Binding Protein Assays (ELPRA) Enable Identification of Bacillus anthracis Colonies
Source: Viruses. 2021 Jul 27;13(8):1462. doi: 10.3390/v13081462 (PMC8402711; doi:10.3390/v13081462)
Supplement: Supplementary file 1 [file viruses-13-01462-s001.zip › viruses-1300262-supplementary.pdf]

Supplementary material for: **Enzyme-linked phage receptor binding protein assay (ELPRA) enables identification of *Bacillus anthracis* colonies**

Peter Braun ‡, Nadja Rupprich ‡, Diana Neif and Gregor Grass *

Bundeswehr Institute of Microbiology (IMB), Dept. of Bacteriology and Toxinology, 80937 Munich, Germany; peter3braun@bundeswehr.org (P.B.); nadjarupprich@googlemail.com (N.R.); diananeif@bundeswehr.org (D.N.); gregorgrass@bundeswehr.org (G.G.)

* Correspondence: gregorgrass@bundeswehr.org; Tel.: +49-99-2692-3981

† Both authors contributed equally to this work.

Supplementary Table S1: Strains, used in this work.

Species	Strain	Characteristics	Reference
B. anthracis	Sterne	pXO1^{+}, pXO2^{-}; host of Gamma phage $^{-}$	[16]
B. anthracis	ATCC4229 (Pasteur)	pXO1^{-}, pXO2^{+}; host of Gamma phage	American Type Culture Collection
B. cereus	ATCC10987	Not a host of Gamma phage	American Type Culture Collection
B. cereus	ATCC4342	Host of Gamma phage	American Type Culture Collection
B. cereus	CDC2000032805	Host of Gamma phage	Centers of Disease Control and Prevention
B. cereus	IMB-4-0-Rott	Not a host of Gamma phage; weak β-hemolysis on blood agar	This work; strain collection of the Bundeswehr Institute of Microbiology
B. cereus s.l.	IMB-2021-1	Host of Gamma phage, no β-hemolysis on blood agar	This work; strain collection of the Bundeswehr Institute of Microbiology

Supplementary Table S2: Oligonucleotide primers used for DNA sequencing and cloning in this work.

**Oligonucleotide**	**Sequence (5'-3')**	**Reference**
27f	AGAGTTTGATCMTGGCTCAG	[21]
341f	CCTACGGGAGGCAGCAG	[22]
534r	ATTACCGCGGCTGCTGG	[22]
1492r	TACGGYTACCTTGTTACGACTT	[21]
NanoLuc foward	AAATGTACAGTCTTCACACTCGAAGATTTCGTT	This study
NanoLuc reverse	AAACTCGAGCGCCAGAATGCGTTCGCA	This study

M = A or C; Y = C or T. Restriction endonuclease recognition sites are underscored.

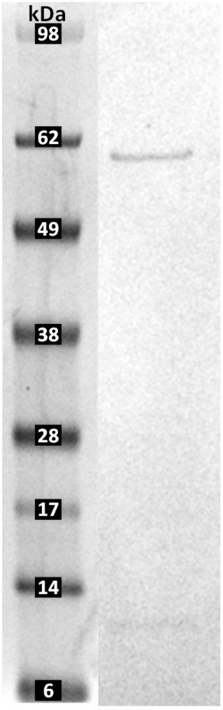

Supplementary Figure S1: SDS-PAGE of heterologously produced NanoLuc-RBP$_{\lambda 03\Delta 1\text{-}120}$-reporter protein. Affinity purified fusion protein was subjected to SDS-PAGE and NanoLuc-RBP$_{\lambda 03\Delta 1\text{-}120}$ protein detected using Imperial™ protein stain (ThermoScientific, Dreieich, Germany). The expected size of the NanoLuc-RBP$_{\lambda 03\Delta 1\text{-}120}$ protein was calculated as 66 kDa. Numbers indicate size-positions of the protein size marker (SeeBlue Plus2 prestained, ThermoFisher Scientific, Darmstadt, Germany).

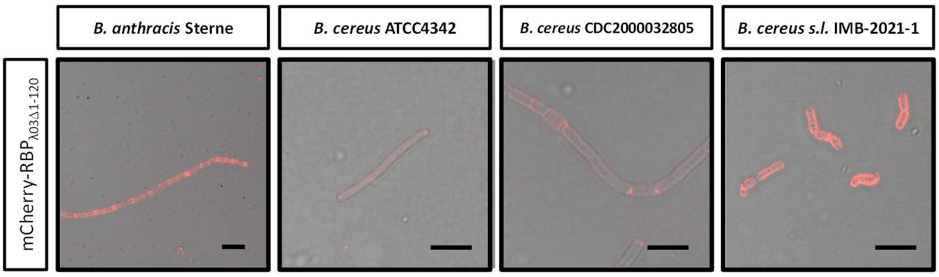

Supplementary Figure S2: Binding of mCherry-RBP$_{\lambda 03\Delta 1\text{-}120}$-reporter to cells of cultures of *B. anthracis* and cross-reacting *B. cereus* cells, respectively. Cultures were grown overnight (16 h) at 37°C with shaking. Cells were labeled (red signals) by adding mCherry-RBP$_{\lambda 03\Delta 1\text{-}120}$ protein, washed to remove unbound RBP and subjected to microscopy. Representative micrographs for labeling of the indicated, *B. cereus* cells are depicted as merged images from light- and fluorescence (excitation: 587 nm, emission: 610 nm) microscopy. Scale bar: 5 µm.

References

16. Sterne M, Proom H Induction of motility and capsulation in *Bacillus anthracis*. J Bacteriol 1957, 74: 541-542.
21. Lane DJ, Pace B, Olsen GJ, Stahl DA, Sogin ML, Pace NR Rapid determination of 16S ribosomal RNA sequences for phylogenetic analyses. Proc Natl Acad Sci U S A 1985, 82: 6955-6959.
22. Watanabe K, Kodama Y, Harayama S Design and evaluation of PCR primers to amplify bacterial 16S ribosomal DNA fragments used for community fingerprinting. Journal of Microbiological Methods 2001, 44: 253-262.
